# Comparison of Phenotypic and Whole-Genome Sequencing-Derived Antimicrobial Resistance Profiles of *Legionella pneumophila* Isolated in England and Wales from 2020 to 2023

**DOI:** 10.3390/antibiotics14101053

**Published:** 2025-10-21

**Authors:** Rediat Tewolde, Rebecca Thombre, Caitlin Farley, Sendurann Nadarajah, Ishrath Khan, Max Sewell, Owen B. Spiller, Baharak Afshar

**Affiliations:** 1 Zoonotic and Acute Respiratory Section, Respiratory and Vaccine Preventable Bacteria Reference Unit, UK Health Security Agency, 61 Colindale Ave, London NW9 5EQ, UK; rebecca.thombre@ukhsa.gov.uk (R.T.); sendurann.nadarajah@ukhsa.gov.uk (S.N.); ishrath.khan@ukhsa.gov.uk (I.K.); spillerb@cardiff.ac.uk (O.B.S.); baharak.afshar@ukhsa.gov.uk (B.A.); 2 Department of Medical Microbiology, Division of Infection and Immunity, School of Medicine, Cardiff University, Cardiff CF14 4XN, UK; farleyc@cardiff.ac.uk (C.F.); sewellm1@cardiff.ac.uk (M.S.)

**Keywords:** whole-genome sequencing, antimicrobial resistance prediction, *Legionella pneumophila*, method validation

## Abstract

**Background:** Antimicrobial resistance (AMR) in *Legionella pneumophila* is emerging as a concern, particularly with resistance to macrolides and fluoroquinolones. Although clinically significant resistance in *Legionella pneumophila* remains uncommon, systematic genomic surveillance using whole-genome sequencing (WGS) is needed to anticipate treatment failure as metagenomic diagnostics move toward routine use. **Objectives:** We assessed the UK Health Security Agency AMR pipeline for predicting resistance in *L. pneumophila* by analysing 522 *L. pneumophila* isolates from England and Wales (2020–2023) together with nine database sequences that carry confirmed 23S rRNA mutations conferring high-level azithromycin resistance. The objective of the present study was to examine the presence of antimicrobial resistance genes (ARGs) in *L. pneumophila* isolates and to determine whether they exhibited phenotypic resistance through minimum inhibitory concentration (MIC) testing. **Methods:** Serogroups (sgs) were determined using an in-house qPCR assay, and *L. pneumophila* non-sg1 isolates were serogrouped using the Dresden monoclonal antibody (mAb) typing method. Sequence types were determined using the standard sequence-based typing method by Sanger sequencing. WGS reads were screened against standard AMR databases to identify resistance genes and resistance-mediating mutations. Agar dilution measured MICs for azithromycin, erythromycin, ampicillin, levofloxacin, tetracycline and spectinomycin in isolates possessing the *bla_OXA-29_*, *lpeAB* or *aph(9)-Ia* gene. **Results:** AMR screening detected *lpeAB*, two allelic β-lactamase variants (*bla_OXA-29_* and *bla_LoxA_*) and *aph(9)-Ia* in 165 of the 522 *L. pneumophila* isolates, while all high-azithromycin MIC reference sequences contained the expected 23S mutation. Only *lpeAB* was associated with a significant twofold elevation in macrolide MICs. Neither β-lactamase variant increased ampicillin MICs, and *aph(9)-Ia* carriage did not correlate with higher spectinomycin MICs. **Conclusions:** Advanced genomic analytics can now deliver timely therapeutic guidance, yet database-flagged genes may not translate into phenotypic resistance. Continuous pairing of curated mutation catalogues with confirmatory testing remains essential for distinguishing clinically actionable determinants such as 23S mutations and *lpeAB* from silent markers like *bla_OXA-29_* and *aph (9)-Ia*.

## 1. Introduction

*Legionella pneumophila* is a Gram-negative environmental bacterium ubiquitously found in water reservoirs and soil [1]. Of its 16 serogroups, serogroup 1 accounts for the majority of human infections. Transmission occurs when aerosolized water containing *L. pneumophila*, commonly from spas, cooling towers or air-conditioning systems, is inhaled; outbreaks are frequently linked to inadequate water-system management [2,3,4].

Upon inhalation, *L. pneumophila* invades and replicates within alveolar macrophages by subverting normal phagolysosomal fusion, precipitating an atypical pneumonia known as Legionnaires’ disease (LD) [5]. Human illness spans a spectrum: Legionnaires’ disease presents as severe, often fatal pneumonia; Pontiac fever manifests as a self-limited, influenza-like respiratory illness; and non-pneumonic legionellosis denotes asymptomatic pulmonary infection confirmed by culture or molecular methods [6,7]. In the UK, case confirmation requires radiological or clinical evidence of pneumonia plus at least one laboratory criterion: culture of *Legionella* from lower respiratory specimens, detection of urinary *L. pneumophila* antigen or PCR detection of *Legionella* DNA in lower respiratory tract samples [8].

First-line therapy for moderate to severe LD comprises macrolides, preferably azithromycin, or respiratory fluoroquinolones (levofloxacin, moxifloxacin), with rifampin plus erythromycin reserved for critical cases [9,10]. Although antimicrobial resistance (AMR) remains uncommon in *L. pneumophila*, sporadic resistant isolates and treatment failures, with mortality rates exceeding 5% despite appropriate therapy, underscore the need for continued surveillance [11].

Early susceptibility tests have reported resistant isolates in US outbreaks. The initial identification of a β-lactamase (*bla_OXA-29_*) in *Legionella gormanii* in 2001 [12] was followed by the homologous *bla_LoxA_* from *L. pneumophila* in 2002 [13]. The RND-family efflux pump *LpeAB* is the most studied resistance gene in *L. pneumophila*, though its clinical significance is debated—some reports find its effect confined to azithromycin [14], whereas others observe elevated minimum inhibitory concentrations (MICs) across all macrolides and report that targeted *lpeAB* deletion reduces MICs by at least fourfold [15]. More recent work shows substantial overlap in macrolide MIC distributions between *lpeAB*-positive and -negative isolates [16,17]. Exceptionally high azithromycin MICs (>2048 mg/L) linked to an A2052G 23S rRNA mutation in separate ST188 strains from France and Belgium emphasize the need for mutation-based surveillance [18,19]. Promoter mutations upstream of *lpeAB* may also drive increased expression [15,20], suggesting that mutations within ARGs may need to be considered. Furthermore, mutations leading to a T83I substitution in the *GyrA* QRDR have been linked to significantly elevated fluoroquinolone MICs [21].

Combined, these findings highlight both the diversity of resistance mechanisms in *L. pneumophila* and the importance of integrating genotypic and phenotypic data to guide therapy. There are several antimicrobial sensitivity testing (AST) methods for *L. pneumophila*; however, the lack of CLSI/EUCAST guidelines and difficulties in *Legionella* culture due to the presence of charcoal in the medium add to the challenges in the detection of minimum inhibitory concentrations (MICs) for *L. pneumophila* [16]. Traditional buffered charcoal yeast extract (BCYE) agar for Legionella culture contains activated charcoal for chelating growth inhibitors, but it also nonspecifically absorbs antibiotics and elevates minimum inhibitory concentrations (MICs). Sewell et al. (2025) note that except for the broth microdilution (BMD) method and LASARUS agar, most methods produce conflicting results for *Legionella* AST [16]. While reports are starting to emerge for *L. pneumophila* that rely solely on genotypic forecasting of resistance [22], this study validate the presence of known antibiotic resistance genes (ARGs) and genomic polymorphisms in key regions of resistance-associated housekeeping genes using whole-genome sequencing (WGS) against the phenotypic measurement of relative antimicrobial susceptibility.

## 2. Results

### 2.1. Source and Epidemiologic Data of Legionella pneumophila Isolates

Between 2020 and 2023, we analyzed 522 *L. pneumophila* isolates, including ten external quality assessment (EQA) environmental samples. Of these, 281 (54%) *L. pneumophila* isolates were from clinical samples and 241 (46%) were from environmental samples. Among the 522 *L. pneumophila* isolates, 457 were sg1 and 65 were non-sg1. Patient metadata extracted from the UKHSA laboratory information management system (LIMS; Supplementary Table S1) showed age recorded for 279 patients, with 13 LD cases (5%) occurring in those aged 16–35 years, 138 (49%) in those aged 36–65 years and 128 (46%) in patients over 65 years. Gender was recorded for 275 patients: 194 (71%) were male and 81 (29%) female, with four cases unreported.

### 2.2. Prevalence of ARGs and Mutations Arising from WGS Analysis

Of the 522 *L. pneumophila* isolates analyzed by WGS, 165 were found to carry one or more ARG or to carry a single nucleotide polymorphism variance from the reference genomes in a housekeeping gene associated with resistance to specific classes of antimicrobials. Table 1 shows the prevalence of listed ARGs *lpeAB* (macrolide efflux pump), *aph(9)-Ia* (spectinomycin-specific aminoglycoside 9-O-phosphotransferase) and *bla_OXA-29_*. None of the isolates were found to carry tet(56) (tetracycline “destructase”), reported in other *Legionella* species. There were also SNPs in the *gyrA* gene that resulted in a conservative amino acid change at position 284 (I248V), and a non-conservative polymorphism in the L4 (*rplD*) ribosomal accessory protein (K185I) was also found. The well-established mutations in the 23S rRNA gene at the macrolide binding site (A2052G) were only found in the sequences of the reference strains with high azithromycin MIC that were downloaded from the ENA database. The genomic data of the reference strains consisted of eight *L. pneumophila* isolates from France and one from Belgium (ENA PRJEB51253 and PRJEB52784).

### 2.3. Phenotypic Antimicrobial Susceptibility Testing Comparison of Isolates

In total, 153 *L. pneumophila* isolates (Table 1) were sent from the Respiratory and Vaccine Preventable Bacteria Reference Unit (RVPBRU), UKHSA, to Cardiff University for antimicrobial sensitivity testing using a previously validated, high-throughput agar-dilution method [23]. Eight *L. pneumophila* isolates were examined by broth microdilution using a recently published, standardized protocol [16]. Reference *L. pneumophila* strains Philadelphia-1 and Knoxville-1 from that protocol were included as controls.

#### 2.3.1. Evaluation of Macrolide Susceptibility

**Figure 1:**
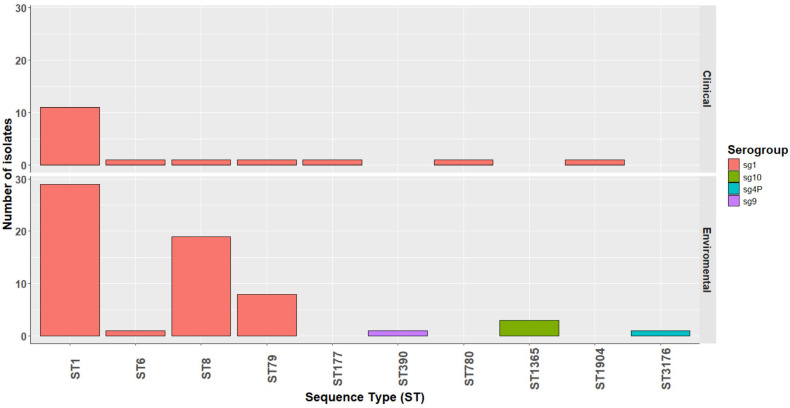
Distribution of *L. pneumophila* isolates carrying the *lpeAB* gene. Categorized by source of isolate (clinical—top, and environmental—bottom), serogroups (sg; see insert legend) and sequence type (ST) on the x-axis.

**Figure 2:**
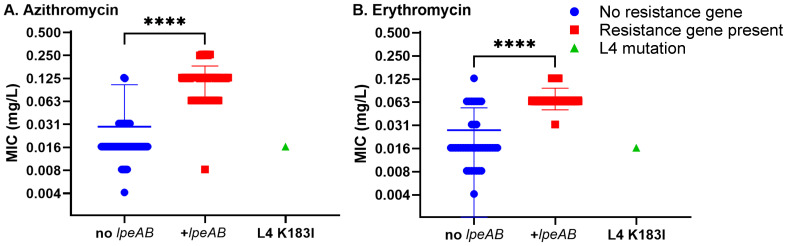
Relative macrolide AST comparison for *L. pneumophila* isolates carrying the *lpeAB* gene or L4 polymorphism. Minimum inhibitory concentrations for azithromycin (**A**) or erythromycin (**B**) for strains carrying *lpeAB* (red squares) or K183I polymorphism in L4 ribosomal accessory protein (green triangle) compared to the rest of the strains that were negative for suspected macrolide resistance determinants (blue circles). Representative data for experiment carried out three times shown. **** represents *p* < 0.0001 by Mann–Whitney U non-parametric test.

Of the 79 *lpeAB*-positive isolates, 76 (96%) belonged to ST1 or closely related STs (differing by one or two alleles): ST1 sg1 (n = 40), ST6 sg1 (n = 2), ST8 sg1 (n = 20), ST79 sg1 (n = 9), ST177 sg1 (n = 1), ST390 sg9 (n = 1) and ST1365 sg10 (n = 3). The remaining three isolates were non-ST1/ST1-like (ST780 sg1, ST1904 sg7 and ST3176 sg4P) (Figure 1). KmerID analysis classified 77/79 (97%) of these *L. pneumophila* isolates as closely related to the *L. pneumophila* Paris reference genome (≥80% similarity). Agar-dilution MICs for azithromycin and erythromycin were significantly higher in *lpeAB*-positive strains than in *lpeAB*-negative strains (*p* < 0.001; Figure 2). However, the overlap in MIC distributions precluded establishing a phenotypic cutoff capable of reliably distinguishing strains on the basis of *lpeAB* carriage. Despite being a non-conservative substitution, L4 K183I was not associated with an increase in macrolide MICs.

Among the 153 *L. pneumophila* isolates, 73 isolates lacked the *lpeAB* gene. Interestingly, two *lpeAB*-negative isolates, 1434081 (ST1358, sg8) and 1456929 (ST47, sg1), exhibited elevated azithromycin MICs of 0.125 and 0.128 mg/L, respectively (Supplementary Table S2). The remaining 71 *lpeAB*-negative isolates displayed the expected low azithromycin MIC range (0.004–0.032 mg/L). The basis for the raised azithromycin MICs in these two *lpeAB*-negative isolates remains unclear. Notably, isolate 1434081 also showed an elevated levofloxacin MIC of 1 mg/L (Supplementary Table S2).

#### 2.3.2. Evaluation of Aminoglycoside Susceptibility

The *aph(9)-Ia* gene encodes an aminoglycoside 9-O-phosphotransferase that specifically phosphorylates spectinomycin, conferring resistance without affecting other aminoglycosides such as kanamycin or streptomycin [24]. Biochemical and structural analyses have confirmed its high substrate specificity when derived from *L. pneumophila*.

**Figure 3:**
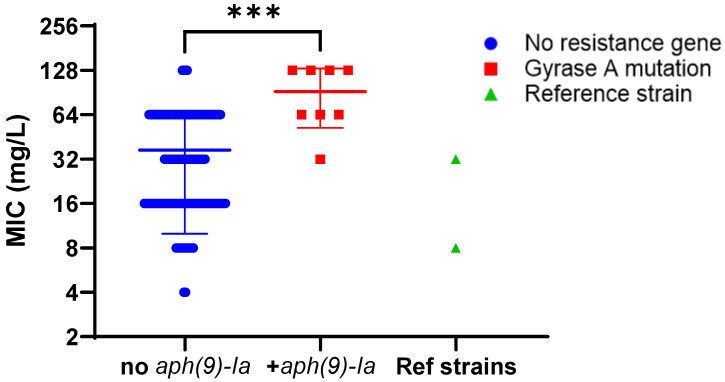
Relative AST comparison for *L. pneumophila* isolates carrying the *aph(9)-Ia* gene. Minimum inhibitory concentrations for spectinomycin for strains carrying *aph(9)-Ia* (red squares) compared to the rest of the strains that were negative for this gene (blue circles) and high-passage reference strains Philadelphia-1 or Knoxville-1 (green triangles), both of which also carry *aph(9)-Ia*. Representative data for experiment carried out three times shown. *** represents *p* < 0.001 by Mann–Whitney U non-parametric test.

Among the 153 *L. pneumophila* isolates, 8 isolates carried *aph(9)-Ia*, all belonging to serogroup 1. 5 isolates were identified as ST82, and the remaining 3 isolates were identified as ST42, ST1522 and ST3134. These eight *L. pneumophila* sg1 isolates exhibited spectinomycin MICs of 32–128 mg/L. While, on average, these isolates had a significantly higher spectinomycin MIC compared to the 145 *aph(9)-Ia*–negative strains, there was considerable overlap in the MIC ranges (Figure 3). There were two isolates without any aminoglycoside resistance genes with MICs of 128 mg/L. Furthermore, we included two reference strains also known to carry *aph(9)-Ia* that had consistently lower MICs—Philadelphia-1 (8 mg/L) and Knoxville-1 (32 mg/L)—suggesting that this gene may not play the dominant role in mediating increased spectinomycin MIC values.

#### 2.3.3. Evaluation of Fluoroquinolone Susceptibility

**Figure 4:**
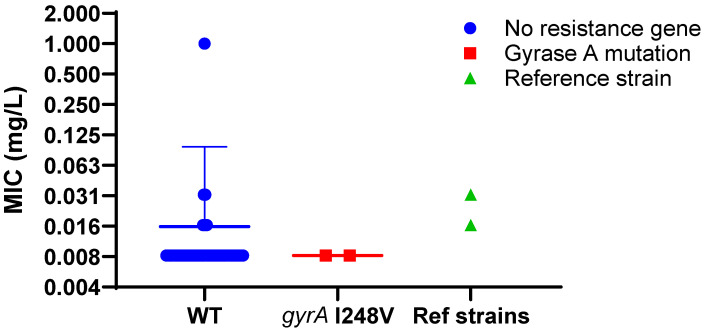
Relative AST comparison for *L. pneumophila* isolates carrying the gyrase A polymorphism. Minimum inhibitory concentrations for levofloxacin for strains carrying the *gyrA* I248V polymorphism (red squares) compared to the rest of the strains that were negative for suspected macrolide resistance determinants (blue circles) or high-passage reference strains Philadelphia-1 or Knoxville-1 (green triangles). Representative data for experiment carried out three times shown.

A conservative substitution in *gyrA* within the quinolone resistance-determining region (QRDR) was identified in two *L. pneumophila* sg1 isolates (ST59 and ST269), replacing isoleucine with valine, and this was classed as a nonsynonymous SNP (amino acid altering). However, as this change preserves the hydrophobic character, it was not expected to confer resistance, and both isolates showed low levofloxacin MICs of 0.008 mg/L, at the lower end of the cohort range (Figure 4; 0.008–0.032 mg/L).

In contrast, isolate 1434081 (ST1358, sg8) exhibited an elevated levofloxacin MIC of 1 mg/L without detectable mutations in *gyrA*, *gyrB* or *parC*. This isolate also showed increased MICs to ampicillin and azithromycin, yet no known resistance genes or target-site mutations explaining these phenotypes were identified.

#### 2.3.4. Evaluation of Tetracycline Susceptibility

No tetracycline resistance genes or mutations were identified in the cohort. Tetracycline MICs for the 153 *L. pneumophila* isolates examined for susceptibility gave a range of 0.5 to 16 mg/L (Supplementary Table S2). While tet(56) is sometimes found in other *Legionella* species, it was not found in the 522 *L. pneumophila* isolates examined by WGS.

#### 2.3.5. Evaluation of β-Lactam Susceptibility

**Figure 5:**
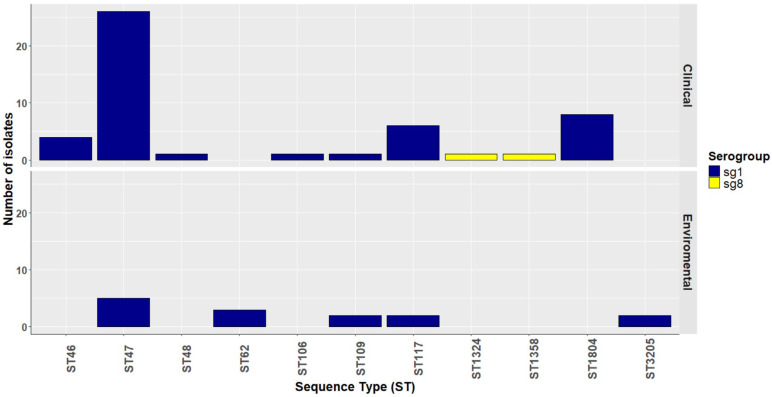
Sequence type distribution of *bla_OXA-29_*-positive isolates in clinical and environmental samples. ST47 isolates carried *bla_OXA-29_* more commonly than other STs in both clinical and environmental samples, of which the majority were sg1 (blue), except for two clinical isolates (sg8) (yellow).

**Figure 6:**
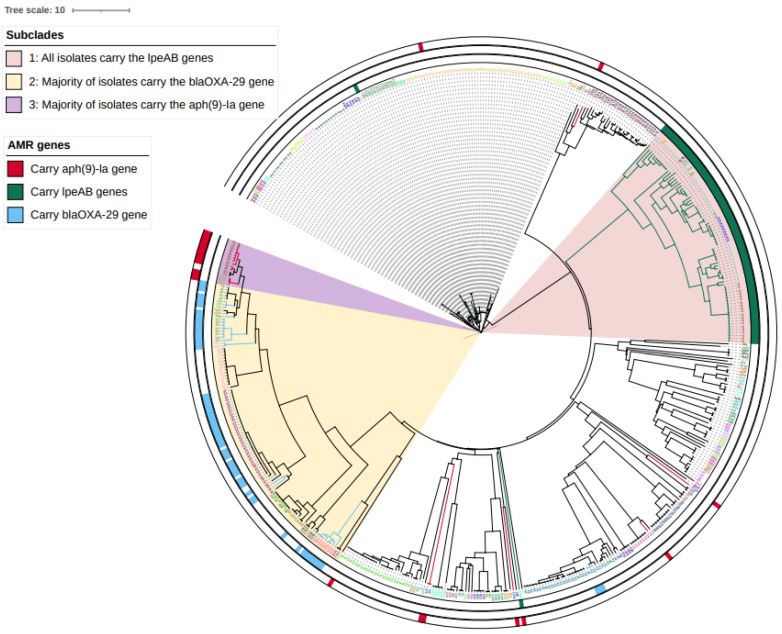
cgMLST neighbour-joining phylogeny of 522 *L. pneumophila* isolates from England and Wales (2020–2023), including both clinical and environmental sources. The tree was inferred using the 50-locus cgMLST scheme. Tips are labelled and colour-coded by sequence type (ST). Coloured annotation strips indicate AMR determinants: green, *lpeAB* (efflux pump); light blue, *blaOXA-29*; red, *aph(9)-Ia*. Visualized in iTOL v6.9.1 (https://itol.embl.de).

Evaluation of β-lactam susceptibility is complicated, as all of the *L. pneumophila* isolates carried the chromosomal class D β-lactamase *bla_LoxA_* [13] in our cohort. However, we did compare ampicillin MICs between isolates with and without the presence of an additional class D β-lactamase gene, *bla_OXA-29_*, originally described in *Legionella* (previously *Fluoribacter*) *gormanii* [12]. *bla_OXA-29_* was detected in 63 of the 522 *L. pneumophila* isolates (49 clinical and 14 environmental) (Figure 5). Of these, 60 isolates were classified as *L. pneumophila* Lorraine-like (≥80% sequence similarity) and clustered within Subclade 2 (Figure 5 and Figure 6), 58 *L. pneumophila* isolates were identified as sg1 and two *L. pneumophila* isolates were identified as sg8, all belonging to ST47 or ST47-like sequence types (one to two allele differences). The remaining three isolates were from the environmental EQA samples, identified as *L. pneumophila* Paris-like (≥80% similarity) sg1 ST62.

**Figure 7:**
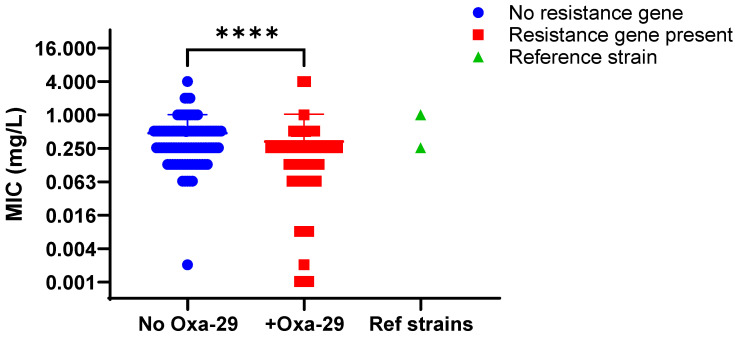
Relative ampicillin MIC comparison for *L. pneumophila* isolates with and without *bla_OXA-29_*. Minimum inhibitory concentrations for ampicillin for strains carrying *bla_OXA-29_* (red squares) compared to the rest of the strains that were negative (blue circles) or high-passage reference strains Philadelphia-1 or Knoxville-1 (green triangles), which also did not carry *bla_OXA-29_*. Representative data for experiment carried out three times shown. **** represents *p* < 0.0001 by Mann–Whitney U non-parametric test.

Contrary to the a priori expectation that bla_OXA-29_ would increase β-lactam MICs, the 63 *bla_OXA-29_*–positive isolates had an overall lower ampicillin MIC relative to the additional isolates not carrying *bla_OXA-29_* (Figure 7; *p* < 0.001) analyzed by the Mann–Whitney U test. Ampicillin MICs for *bla_OXA-29_* carriers ranged from 0.002 to >2 mg/L, which was similar to the range observed in *bla_OXA-29_*_-_negative isolates (0.002 to >2 mg/L) (Supplementary Table S2). These data provide no evidence that *bla_OXA-29_* elevates β-lactam MICs in *L. pneumophila* and suggest lineage-associated or other confounding factors may underlie the observed distribution.

### 2.4. Genomic Analysis of L. pneumophila Isolates

#### 2.4.1. Detection of 23S rRNA Mutations in High-Azithromycin MIC Isolates

While no high macrolide MIC isolates were available in our cohort of 522 *L. pneumophila* isolates, we have previously shown that high macrolide MIC ST188 *L. pneumophila* isolates from France and Belgium [16,19] had azithromycin MICs ≥ 2048 mg/L. To validate the GeneFinder AMR software (version 2-7), nine publicly available genomes for these isolates were analyzed, and all were identified as carrying the A2052G mutation in the 23S rRNA.

#### 2.4.2. cgMLST-Based Core-Genome Phylogenetic Analysis

A total of 522 *L. pneumophila* isolates (281 clinical, 241 environmental from England and Wales from 2020 to 2023) were typed by cgMLST, and a neighbour-joining phylogeny was generated. Ninety-eight sequence types (STs) were identified; tree tips were labelled and colour-coded by ST (Figure 6).

**Figure 8:**
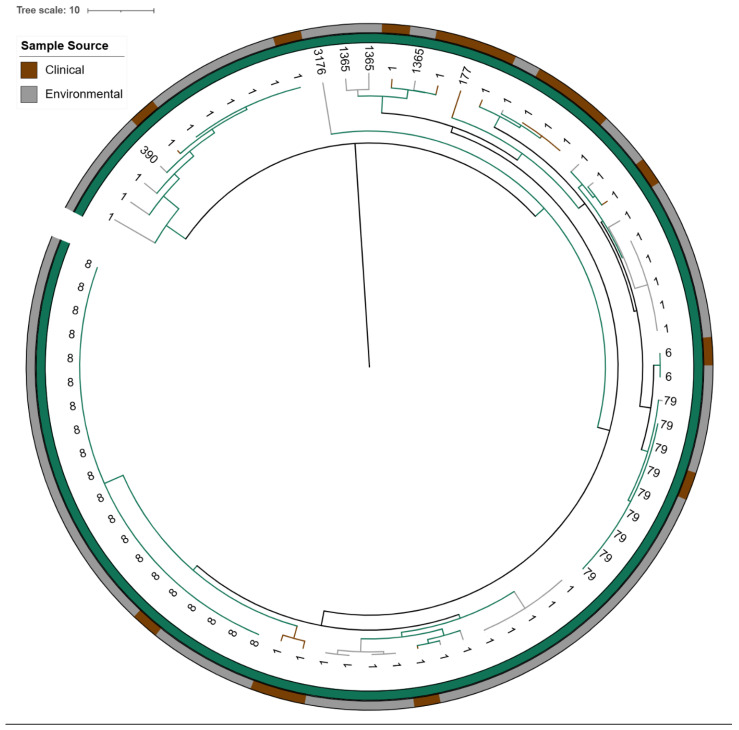
Neighbour-joining phylogeny of Subclade 1 (*L. pneumophila* Paris lineage, *lpeAB*-positive). Tips are labelled and colour-coded by sequence type (ST). All isolates in Subclade 1 are identified as *L. pneumophila* Paris with most assigned to ST1, ST8, ST6 or ST79 and carrying *lpeAB gene*. Green annotation strips indicate the presence of the macrolide resistance-associated *lpeAB* efflux operon. Annotation strips in brown and grey indicate the source of each isolate, representing clinical and environmental origins, respectively. Visualized in iTOL v6.9.1. (https://itol.embl.de).

**Figure 9:**
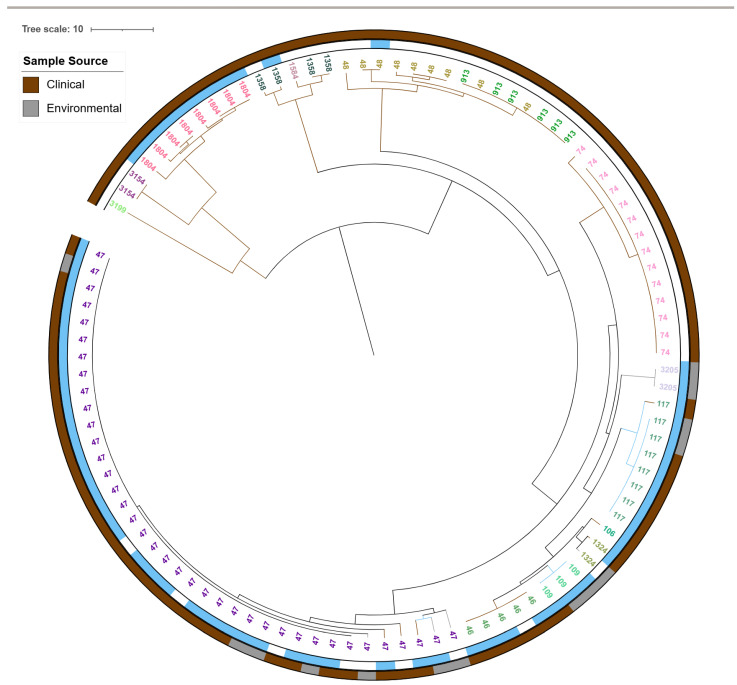
Neighbour-joining phylogeny of Subclade 2 (*L. pneumophila* Lorraine lineage, predominantly *bla_OXA-29_*-positive). Tips are labelled and colour-coded by sequence type (ST). All isolates are identified as closely related to the *L. pneumophila* Lorraine strain, with most assigned to ST46, ST47, ST109, ST117, ST1804, or ST3205 and carrying *bla_OXA-29_*. Blue annotation strips indicate the presence of the β-lactam resistance-associated gene *bla_OXA-29_*. Annotation strips in brown and grey indicate the source of each isolate, representing clinical and environmental origins, respectively. Visualized in iTOL v6.9.1. (https://itol.embl.de).

Isolates sharing STs and those harbouring *lpeAB*, *bla_OXA-29_* or *aph(9)-Ia* clustered predominantly within Subclades 1–3 (Figure 6). Subclade 1 comprised the *L. pneumophila* Paris-like lineage, all ST1 or ST1-like, and all carried *lpeAB* (Figure 6 and Figure 8). Subclade 2 comprised the *L. pneumophila* Lorraine-like lineage, all ST47 or ST47-like and majority of the isolates carried *bla_OXA-29_*. Subclade 3 also comprised the *L. pneumophila* Lorraine-like lineage, including ST82 and ST1522, and majority of the isolates carried *aph (9)-Ia* (Figure 9).

## 3. Discussion

Enhanced AMR genomic surveillance using WGS may evolve to become essential for the early detection of resistance in *L. pneumophila* and for informing therapy, particularly with the growing use of direct metagenomic diagnostics. Patient metadata extracted for *L. pneumophila* isolates from the UKHSA laboratory information management system (LIMS) indicated that infections mostly affected males, and in individuals aged 36–65 years, infections were only marginally more common than in those over 65.

In the 522 *L. pneumophila* isolates from England and Wales (2020 to 2023), WGS screening identified *lpeAB* (macrolide efflux), *bla_OXA-29_* (class D β-lactamases) and *aph(9)-Ia* (spectinomycin phosphotransferase). No tetracycline or fluoroquinolone resistance genes and no quinolone resistance-determining region mutations associated with resistance were detected.

Genotype–phenotype comparisons showed that *lpeAB* carriage was associated with higher azithromycin and erythromycin MICs, although MIC distributions overlapped with *lpeAB*-negative isolates, which prevents a discriminatory clinical threshold. Two *lpeAB*-negative clinical isolates displayed modestly elevated azithromycin MICs, indicating that factors other than *lpeAB* can shift macrolide MICs. These findings align with reports that *lpeAB* upregulation can follow promoter mutations or sub-inhibitory macrolide exposure and confers only moderate increases in macrolide MICs, with debated clinical significance [14,15,16,17,20].

In contrast to expectations for a β-lactamase, *bla_OXA-29_* was not associated with increased ampicillin MICs. MIC ranges were similar in *bla_OXA-29_*-positive and -negative groups, and the *bla_OXA-29_* group showed lower ampicillin MICs in Mann–Whitney testing. All *L. pneumophila* isolates tested carried chromosomal *bla_LoxA_* [13], and *bla_OXA-29_* occurred almost exclusively in the Lorraine lineage (ST47/ST47-like). These data support prior observations that *bla_OXA-29_* detection does not necessarily translate into β-lactam resistance in *L. pneumophila* [9,12,25] and suggest that lineage, rather than enzyme activity, explains the observed distribution. It is known that plasmids can rapidly disseminate ARGs, like those that code for β-lactamase in many bacterial populations, and these can be acquired from or spread to environmental bacterial communities [26]. However, evidence of plasmid-mediated transfer of β-lactamase genes in *Legionella* is limited. Short-read assemblies make it challenging to determine gene locations with certainty, and it is ideal to use long-read sequencing to define whether *bla_OXA-29_* is plasmid-borne and to assess its mobilization potential, as plasmid-encoded oxacillinase enzymes can disseminate in healthcare and environmental settings.

It is important to note, however, that as *L. pneumophila* is an intracellular pathogen, beta-lactams could not be used for therapeutic purposes in Legionellosis. Resistance testing for beta-lactams in Legionella has limited clinical relevance since these antibiotics are not considered therapeutic options for Legionella infections These observations are more important for ARG mobility and distribution surveillance.

For spectinomycin, *aph(9)-Ia* was detected in a subset of *L. pneumophila* isolates, but MICs overlapped those of *aph(9)-Ia*-negative strains despite having previously been identified as a resistance mechanism in earlier reports [24,27]. Clearly, other mechanisms are responsible for elevating spectinomycin MICs in both *aph(9)-Ia*-positive and -negative isolates, but an underlying gene or mutation has not yet been identified. No other spectinomycin ARGs, such as the *ant-(9)* adenyltransferases described in other bacteria [28], were present in the WGS for our cohort.

High-level macrolide resistance mediated by 23S rRNA mutations was not identified in these *L. pneumophila* isolates. However, the UKHSA pipeline correctly identified the A2052G mutation in nine externally available sequences for the ST188 isolates from France and Belgium that exhibit azithromycin MICs above 1000 mg/L, confirming its ability to detect clinically relevant resistance mutations [18,19]. Together, these data reinforce that WGS is most actionable for validated resistance-mediating mutations, while database hits for certain genes may not predict phenotype.

We acknowledge some limitations in our study. It is challenging to determine the genomic context of *bla_OXA-29_* using short-read WGS alone to reliably resolve chromosomal versus plasmid location; hybrid assemblies with long reads (e.g., ONT) are needed to establish mobility potential. We did not quantify gene expression or copy number, so regulatory effects that modulate phenotype were not assessed [29]. No induction experiments were performed; therefore, we did not test whether sub-inhibitory exposure or environmental cues can upregulate identified genes and alter MICs. The observation that *aph(9)-Ia* and *bla_OXA-29_* were present without corresponding MIC elevation, whereas *lpeAB* was associated with only modest, overlapping MIC shifts, suggests that gene presence alone is an unreliable predictor of resistance in *L. pneumophila* under current conditions [17]. Further work should pair phenotyping with expression analyses and long-read sequencing to resolve the gene context and inducibility.

In summary, *lpeAB* confers a modest elevation in macrolide MICs without a clear phenotypic cutoff, *bla_OXA-29_* does not raise β-lactam MICs and *aph(9)-Ia* does not explain spectinomycin MIC variation in this collection. Mutation-based detection, particularly in 23S rRNA for macrolides, remains the strongest genomic predictor of clinically significant resistance. Future work should pair WGS with long-read sequencing to establish the gene context, include expression analyses for contentious loci and continue contemporaneous MIC testing to refine genotype–phenotype inference.

## 4. Materials and Methods

### 4.1. Bacterial Strains and Culture Conditions

A panel of 522 *L. pneumophila* isolates were collected from clinical and environmental samples from England and Wales between 2020 and 2023 at the Zoonotic and Acute Respiratory Section (ZARS), within the Respiratory and Vaccine Preventable Bacteria Reference Unit (RVPBRU), UK Health Security Agency (UKHSA). All *L. pneumophila* isolates tested in this study were initially identified by an in-house qPCR assay targeting the mip (*L. pneumophila*-specific target) and wzm [30]. These *L. pneumophila* isolates were sequence typed using the standard sequence-based typing (SBT) method by Sanger sequencing [31]. The *L. pneumophila* non-sg1 isolates were serogrouped using the Dresden mAb typing method [32]. 

For whole-genome sequencing (WGS), pure cultures (from a single colony) were recovered from frozen beads on BCYE agar plates and incubated at 37 °C for 2–10 days until colonies appeared. DNA was extracted from RNase-treated lysates (ATL buffer) using the QIAsymphony kit and the QIAsymphony platform (Qiagen, Germantown, MD, USA). Sanger sequencing and WGS were performed at the Colindale Sequencing Laboratory (CSL), UKHSA.

### 4.2. Illumina Sequencing and Read Preparation

Libraries were prepared with Nextera XT (Illumina, San Diego, CA, USA) and sequenced on an Illumina NextSeq 1000 100 bp read size. Demultiplexing used CASAVA v1.8.2. Reads were trimmed with Trimmomatic [33] to remove low-quality bases (PHRED < 30) from read ends.

### 4.3. FASTQ Quality Thresholds

Only datasets passing predefined QC were analyzed. After trimming, read pairs < 50 bp were discarded. Samples with a total post-trim yield < 150 Mb were excluded.

### 4.4. Legionella Species Identification

Species ID and contamination screening was performed using the validated UKHSA k-mer tool; KmerID (version 0.1) (https://github.com/phe-bioinformatics/kmerid, accessed on 4 September 2024. Identity was accepted at ≥80% similarity based on whole-genome comparison; mixed/contaminated read sets were excluded.

### 4.5. AMR Detection in Isolates from England and Wales

AMR determinants were called with GeneFinder (version 2-7) (https://github.com/ukhsa-collaboration/gene_finder (accessed on 4 September 2024)). Gene hits also required 100% breadth of coverage and ≥85% nucleotide identity relative to the reference β-lactamase (*bla_LoxA_*) gene.

Gene hits also required 100% breadth of coverage and ≥90% nucleotide identity relative to the reference. Targets included the following:

(i) macrolide determinants: *lpeAB* (efflux; *lpeA*, *lpeB*), 23S rRNA methylases *erm(A)*, *erm(B)*, *erm(C)*, *erm(F)*, phosphotransferase *mph(A)* and esterases *ere(A)*, *ere(B)*;
(ii) class D β-lactamase *bla_OXA-29_*;
(iii) tetracycline destructase *tet(56)*;
(iv) spectinomycin phosphotransferase *aph(9)-Ia*.

GeneFinder was also used to screen for point mutations associated with resistance in 23S rRNA (*rrl*; G2051A, A2052T/C/G, A2053C/G, C2605A/G/T), *gyrA* (G242C, C248T, G259A), *gyrB* (C1279A, G1391A), *parC* (G251A, G253C, A254C), *rplD* (L4; A185G, A188C, G190C/G, G191A/G, C194A, G196C/T, G197A/C) and *rplV* (L22; C260T, A269T, G272A).

### 4.6. AMR Detection in External Macrolide-Resistant Isolates

Nine highly macrolide-resistant *L. pneumophila* ST188 isolates (eight isolates from France and one isolate from Belgium; ENA PRJEB51253 and PRJEB52784) were downloaded and analyzed with GeneFinder to detect the A2052G mutation in 23S rRNA [18,19].

### 4.7. Minimum Inhibitory Concentrations (MICs)

Of the 522 *L. pneumophila* isolates, 165 harboured one or more AMR determinants; 153 *L. pneumophila* isolates were selected for MIC testing (Table 1). Reference strains *L. pneumophila* Philadelphia-1 (NCTC 11192) and Knoxville-1 (NCTC 11286) were included. MICs for erythromycin, azithromycin, levofloxacin, spectinomycin and doxycycline were determined using the LASARUS agar-dilution method [23]. Where specified, a subset was tested by broth microdilution using a standardized protocol [16].

### 4.8. Statistical Analysis

Analyses were performed in GraphPad Prism (version 10.5.0 GraphPad Software). Non-parametric tests were used where appropriate; exact tests and *p*-values are reported in figure legends.

### 4.9. Genome Assembly

Reads were assembled de novo with SPAdes [34] with the following parameters: ‘spades.py–careful -1 strain.1.fastq.gz -2 strain.2. fastq-t 2-k 21,33,55,77. All the contigs were checked for quality. Contigs < 500 bp were removed. Assembly quality was assessed with QUAST v5.2.0 [35].

### 4.10. Core-Genome MLST and Phylogeny

cgMLST was performed with chewBBACA (https://github.com/B-UMMI/chewBBACA (version v3.4.2; accessed on 6 September 2025) using a 50-locus scheme [36] Allele calls (results_alleles.tsv) were used to build a neighbour-joining tree in GrapeTree (RapidNJ). Trees were visualized in iTOL v6.9.1.

### 4.11. Data Deposition

Sequence data for the 153 newly sequenced *L. pneumophila* genomes have been deposited in ENA under accession PRJNA1307548.

## 5. Conclusions

In the present study, WGS-based AMR surveillance was performed across 522 clinical and environmental isolates of *L. pneumophila* to investigate the presence of antimicrobial resistance genes (ARGs) and to determine whether the presence of these genes resulted in phenotypic resistance through minimum inhibitory concentration (MIC) testing. ARGs such as *bla_OXA-29_* and *aph(9)-Ia* were detected in the *L. pneumophila* isolates; however, the presence of these genes did not raise the MIC. The macrolide resistance gene *lpeAB* was detected in some of the isolates, and it was associated with only a modest, statistically significant increase in macrolide MICs without a usable phenotypic cutoff. High-level macrolide resistance driven by point mutations in the 23S rRNA (A2052G) was absent in the *L. pneumophila* isolates from England and Wales. The WGS pipeline correctly identified the point mutations in external *L. pneumophila* ST188 controls, confirming pipeline accuracy for mutation-based resistance. The *bla_OXA-29_* in the Lorraine lineage did not elevate β-lactam MICs, reinforcing the view that gene presence alone is a poor predictor of phenotype in *L. pneumophila*. Given selection pressures from sub-inhibitory antibiotics, biocides and heavy metals in environmental reservoirs, routine combined genotypic and phenotypic surveillance remains essential. Priorities include hybrid long-read assemblies to resolve the gene context and induction/expression studies, alongside integration with metagenomic diagnostics, to detect emerging resistance early and guide stewardship and environmental control.

## Figures and Tables

**Table 1:** Prevalence of known antimicrobial resistance genes or housekeeping gene mutations.

ARG or Mutation	Found in N Isolates	Phenotypically Tested
*lpeAB*	79	79
*aph(9)-Ia*	22	8
*bla_OXA-29_*	63	63
*gyrA* I248V	2	2
L4 K183I	1	1

## Data Availability

The original contributions presented in this study are included in the article and Supplementary Materials. Further inquiries can be directed to the corresponding author.

## Supplementary Materials

The following supporting information can be downloaded at: https://www.mdpi.com/article/10.3390/antibiotics14101053/s1, Table S1: Metadata and WGS results; Table S2: MIC after 72HR.
